# Molecular Cooperation of Ion‐Free Ternary Complexes Enhances Efficiency and Stability of Perovskite Solar Cells

**DOI:** 10.1002/smsc.202300165

**Published:** 2023-11-27

**Authors:** Peiyao Dong, Xuejiao Wu, Li Yang, Jinbao Zhang

**Affiliations:** ^1^ College of Materials Fujian Key Laboratory of Advanced Materials Xiamen Key Laboratory of Electronic Ceramic Materials and Devices Xiamen University Xiamen 361005 China; ^2^ Shenzhen Research Institute of Xiamen University Shenzhen 518000 China

**Keywords:** chemical dopings, hole-transport layers, perovskite solar cells, stabilities, ternary complexes

## Abstract

Chemical dopants such as ionic additives are essential in hole‐transport layers (HTLs) to enhance their charge‐transport abilities in perovskite solar cells (PSCs). However, these ionic components often cause issues of ion migration and phase segregation, which limit the reliability and stability of PSCs. Herein, an effective strategy of cooperative ternary components (CTC) is developed to enhance device efficiency and stability by combining molecular additives and conjugated polymers into the conventional 2,2′,7,7′‐tetrakis (N,N‐di‐p‐methoxyphenyl‐amine) 9,9′‐spirobifluorene (Spiro)‐based HTLs. All‐molecule‐based CTC enables negligible phase segregation in the HTL compared to ionic doping. The collaborative roles of small molecules and conducting polymer in the CTC blend facilitate to enhance the interfacial charge extraction and the charge transport in the bulk HTL. Consequently, the CTC strategy contributes to a substantial increase of the device efficiency from 14.97% (the control) to 20.14% (CTC), which is among the highest reported efficiency for the ion‐free spiro‐based PSCs. More encouragingly, the CTC‐based devices demonstrate remarkable environmental stability, maintaining 90% of the initial efficiency after about 2500 h under ambient conditions without any encapsulation. In this work, a novel doping approach is provided for the fabrication of stable and efficient PSCs.

## Graphical abstract

An effective strategy of cooperative ternary components is developed to enhance device efficiency and stability by combining molecular additives and conjugated polymer into the conventional 2,2′,7,7′‐tetrakis (N, N‐di‐p‐methoxyphenyl‐amine) 9, 9′‐spirobifluorene–based hole‐transport layers.

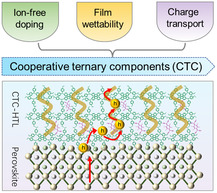

## 1 Introduction

Semiconductors have shown tremendous application potentials in electronic devices, e.g., light‐emitting diodes (LEDs), solar cells, and organic transistors,^[^ ^1^, ^2^, ^3^ ^]^ due to their benefits in terms of ease of solution processing, low manufacturing cost, and high flexibility.^[^ ^4^, ^5^, ^6^ ^]^ A variety of strategies such as chemical doping and structural modification have been proposed to modulate their electrical conductivity, and to further boost the performance of organic semiconductors in optoelectronic devices.^[^ ^7^, ^8^ ^]^

In n–*i*–p perovskite solar cells (PSCs), the hole‐transport layer (HTL) commonly used is a small molecule called 2,2′,7,7′‐tetrakis (N, N‐di‐p‐methoxyphenyl‐amine) 9, 9′‐spirobifluorene (Spiro‐OMeTAD–Spiro). The HTL plays a crucial role in preventing contact between the perovskite and the Ag electrode as well as keeping moisture from entering the perovskite layer.^[^ ^9^, ^10^ ^]^ However, the pristine Spiro film exhibits low intrinsic conductivity, which limits both charge‐collection efficiency and transport capacity.^[^ ^11^ ^]^ Presently, lithium bis((trifluoromethyl)sulfonyl)azanide (LiTFSI) and 4‐tert‐butylpyridine (TBP) are the most commonly used dopant combination in Spiro‐based HTLs to achieve satisfactory conductivity (3 × 10^−5^ S cm^−1^), resulting in encouraging power conversion efficiencies (PCEs) of more than 25% in n–*i*–p PSCs.^[^ ^12^, ^13^ ^]^ The anion in the lithium salts could affect the work function of HTL by regulating the energetics of the hole polarons, and also modulate the film conductivity,^[^ ^14^ ^]^ whereas the cation Li^+^ could participates in the oxidation of the Spiro and decreases the barrier of carrier transportation. Previous reports have also proved that the cation could improve the interfacial carrier‐extraction properties.^[^ ^15^, ^16^, ^17^ ^]^ However, recent studies indicated that the devices suffer from severe stability issues as a result of the hygroscopic nature and ion migration of LiTFSI.^[^ ^18^ ^]^ Meanwhile, the voltaic TBP, which is responsible for improving film uniformity, further damaged the perovskite layer by forming a PbI_2_‐TBP heterophase and promoted the de‐doping of Spiro.^[^ ^19^, ^20^, ^21^, ^22^ ^]^ Therefore, the development of alternative additives to increase the device stability is an effective strategy, such as hydrophobic lithium salts, complexes which interact with Li^+^ and Li‐free salts.^[^ ^23^, ^24^, ^25^ ^]^ Alternatively, some efforts have been devoted to develop dopant‐free organic molecules and polymers for PSCs,^[^ ^26^ ^]^ and however, they showed either relatively low performance or tedious synthesis, which are not feasible for scalable applications. To optimize the electrical properties of Spiro, our group recently applied thermal evaporation method to deposit dopant‐free HTL with controlled thickness,^[^ ^27^ ^]^ but the devices suffered from strong light‐soaking effects, which were related to the poor electrical conductivity of undoped Spiro.^[^ ^28^ ^]^ Therefore, challenges remain in developing reliable HTLs to achieve efficient and stable PSCs.

In contrast to these ionic additives, hydrophobic molecules have been proved to be more suitable as the p‐type additives to improve the moisture resistance of PSCs. The molecular dopant 2,3,5,6‐tetrafluoro‐7,7,8,8‐tetracyanoquinodimethane (F4TCNQ) is a promising oxidant due to its deeper lowest unoccupied molecular orbital (LUMO) level, which matches well with the highest occupied molecular orbital (HOMO) level of many donors.^[^ ^29^ ^]^ Additionally, the hydrophobicity and nonionic property of F4TCNQ is also beneficial for the device stability.^[^ ^30^ ^]^ For example, F4TCNQ has been previously introduced in the HTLs based on poly(3‐hexylthiophene‐2,5‐diyl) (P3HT) and Spiro,^[^ ^31^, ^32^ ^]^ to optimize the device performance, but their PCEs are still lagging behind the ion‐doped HTLs, and the individual and cooperative roles of these molecules in PSCs have not been well understood.^[^ ^33^ ^]^

In this respect, we rationally design a cooperative ternary components (CTC) approach to investigate the fundamental roles of individual additives and to further boost the photovoltaic performance of PSCs. Specifically, we introduce the hydrophobic F4TCNQ as molecular dopant to the Spiro‐based HTL, aiming to improve the doping level of Spiro as well as the environmental stability of devices. To further increase the charge‐transport property in the bulk HTL, the conducting polymer P3HT is incorporated into to the film. The CTC strategy enables the fabrication of ion‐free HTLs based on Spiro + F4TCNQ + P3HT for n–*i*–p PSCs. The results show that F4TCNQ enables the simultaneous doping of Spiro and P3HT, which promotes high carrier density and charge conductivity in the HTLs. The small‐size Spiro favors the interfacial hole transfer from perovskite toward HTLs, and the long‐chain P3HT guarantees sufficient intramolecular charge transport. All of these properties contribute to significant suppression of non‐radiative recombination and carries losses at interfaces, leading to dramatic improvement in device performance. As a result, the device based on CTC exhibits an increased PCE of 20.14% compared to 14.97% for the control, which represents the record efficiency for ion‐free Spiro‐based HTLs in PSCs. More importantly, the corresponding devices demonstrate remarkable environmental stability by retaining 90% of PCE after 2500 h in an ambient environment. This work reveals the individual and comprehensive roles of these molecular additives in the HTLs, and provides a novel molecular doping strategy of Spiro HTLs for obtaining PSCs with both high efficiency and stability.

## 2 Results and Discussion

**Figure 1:**
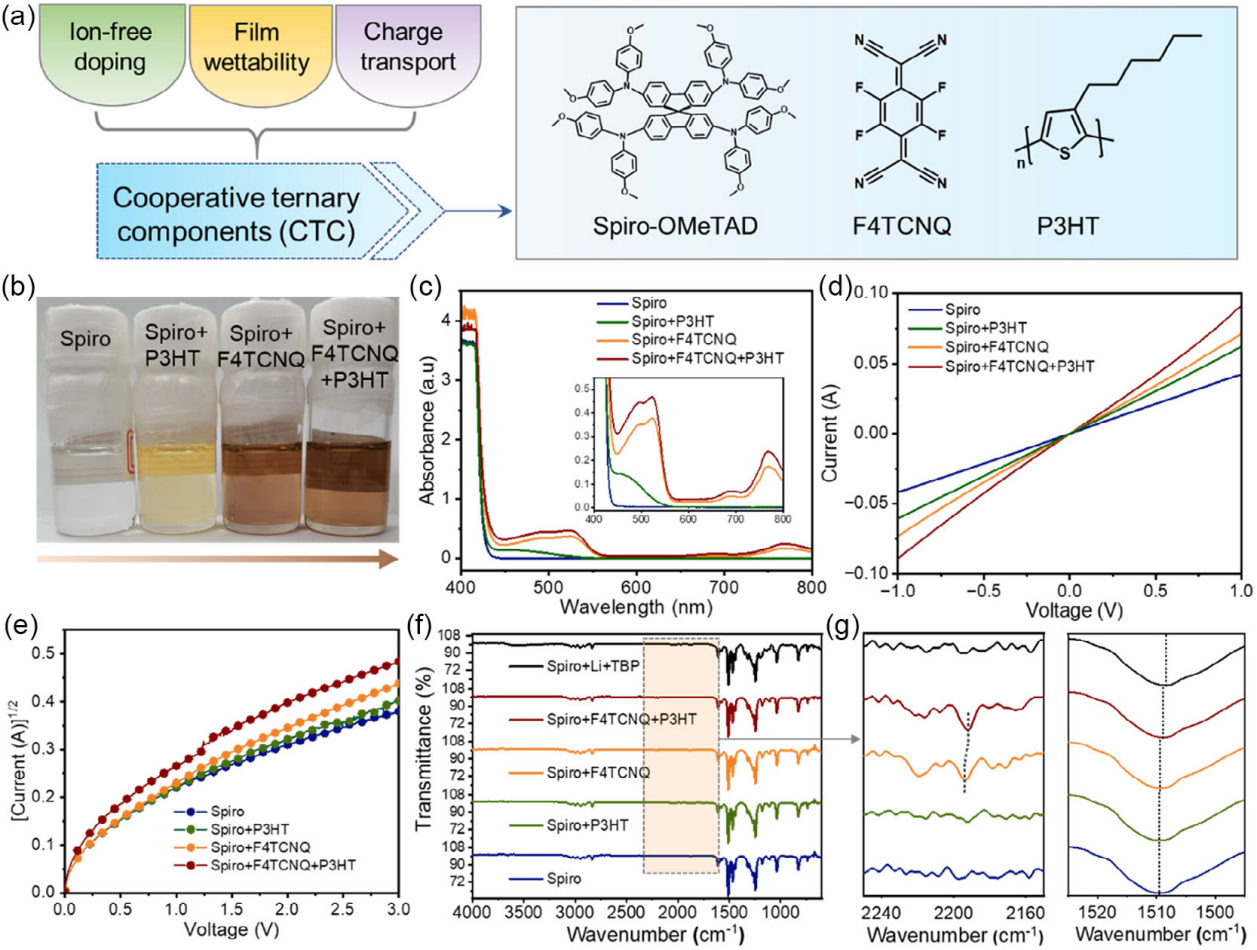
a) Molecular structures of 2,2′,7,7′‐tetrakis (N, N‐di‐p‐methoxyphenyl‐amine) 9, 9′‐spirobifluorene (Spiro‐OMeTAD—Spiro), 2,3,5,6‐tetrafluoro‐7,7,8,8‐tetracyanoquinodimethane (F4TCNQ), and poly(3‐hexylthiophene‐2,5‐diyl) (P3HT). b) Digital photographs and c) UV–vis absorption spectra of the Spiro solution with different molecular additives. d) Current–voltage (*I–V*) curves, e) space‐charge limited current tests, and f,g) Fourier transform infrared spectra of the Spiro films with different molecular additives.

To investigate the fundamental roles that traditional ionic dopants and molecular dopants play in the optoelectronic performance of Spiro, we prepared various samples by combining distinct molecules in the HTLs, namely Spiro + Li + TBP, Spiro, Spiro + P3HT, Spiro + F4TCNQ, and Spiro + F4TCNQ + P3HT, respectively. **Figure** **1**a contains the molecular structures for Spiro, F4TCNQ, and P3HT. We first examined the ultraviolet–visible (UV–vis) absorption spectra of these samples, as shown in Figure 1c and S1, Supporting Information. The absorbance at about 525 and 770 nm was assigned to the typical peaks of Spiro^+^,^[^ ^34^ ^]^ and the ionized F4TCNQ.^[^ ^35^ ^]^ Since the concentration of the Spiro solution and the dilution ratio were kept the same, the content of Spiro^+^ could reflect the p‐doping yield. By monitoring the evolution of the Spiro^+^ peak, a gradual enhancement of the absorption intensity was obtained after introducing F4TCNQ, indicating that F4TCNQ, as an effective electron acceptor, could promote the oxidation efficiency of Spiro. In addition, the p‐doping yield increased when additional P3HT was added, which is in accordance with the darker color of the solution as shown in Figure 1b. The increase of p‐doping yield could be due to the chemical reaction between P3HT and F4TCNQ, in which more ionized F4TCNQ was generated for coupling with the radical dipoles.^[^ ^36^ ^]^

Higher p‐doping yield favors the formation of a larger amount of charge carriers in the molecular‐doped film. To reveal the effect of doping level on the conductivity of HTLs, the current–voltage (*I*–*V*) curves of different samples were obtained with an indium tin oxides (ITO)/Spiro/Ag device structure. As illustrated in Figure 1d, the conductivity of Spiro + F4TCNQ + P3HT is significantly greater than that of Spiro + F4TCNQ, Spiro + P3HT, and Spiro. Given that a larger amount of charge carriers was formed in the sample of Spiro + F4TCNQ, we suggest that the relatively low charge mobility is the factor limiting the conductivity of the HTL based on Spiro + F4TCNQ. This result could indicate that the major role of P3HT is to improve the carrier mobility due to faster intramolecular charge transport,^[^ ^37^, ^38^ ^]^ compared to small‐molecule Spiro.^[^ ^39^ ^]^ This hypothesis was confirmed by the space‐charge limited current tests (Figure 1e), showing that the charge mobility of the film was furtherly improved with the addition of P3HT. Figure S2, Supporting Information, exhibits the comparison of the charge mobility for molecular HTLs and the traditionally doped HTL with Spiro + Li + TBP, showing that the ion‐doped film has higher charge mobility than the molecular‐doped one, which could further imply the importance of counter ions in the charge transport.^[^ ^40^ ^]^

To further investigate the feedback effect of F4TCNQ and P3HT on Spiro, the Fourier transform infrared spectroscopy of different Spiro films was performed (Figure 1f). The absorption peak at about 2230 cm^−1^ of the cyano bond (C≡N) is an indicator of the existence of charges on the F4TCNQ molecule (Figure 1g).^[^ ^33^, ^41^ ^]^ The shift of C≡N peak indicates the formation of the F4TCNQ anion radical state after accepting electrons from Spiro and P3HT. Similar phenomena were also observed when P3HT was mixed with F4TCNQ (Figure S3, Supporting Information). The addition of P3HT in Spiro‐based HTL could facilitate the hole extraction and transport compared to the bare Spiro, considering the fact that the doped P3HT by F4TCNQ generate new pathways for carrier transport.^[^ ^42^ ^]^ Furthermore, the peak at 1510 cm^−1^ observed in the Spiro samples can be ascribed to the C—N stretching mode of the triphenylamine group.^[^ ^43^ ^]^ The peak for Spiro+Li+TBP was redshifted by about 1.2 cm^−1^, proving the electrostatic interaction between the radical dipoles and the anions, which could probably lead to efficient charge extraction and transport.^[^ ^44^ ^]^ In contrast to the inconspicuous shift for F4TCNQ‐doped Spiro samples, the notable peak shift in the film of Spiro + F4TCNQ + P3HT suggests the reaction between P3HT, F4TCNQ, and Spiro. The spectroscopic analysis reveals that effective p‐type doping was realized when P3HT and F4TCNQ were simultaneously mixed with Spiro, thereby generating more radicals and increasing the carrier‐transport capability of ion‐free Spiro HTLs.

**Figure 2:**
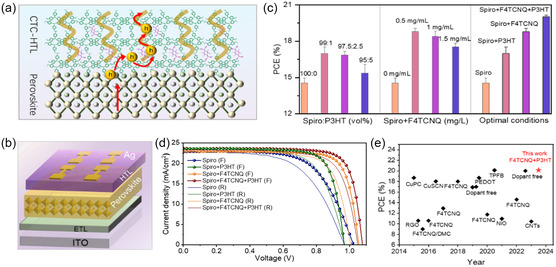
a) The schematic representation of charge‐transport mechanism based on Spiro + F4TCNQ + P3HT components. b) Scheme of n–*i*–p device structure of perovskite solar cells (PSCs). c) The statistical distribution of power conversion efficiencies for devices with different hole‐transport layers (HTLs). d) Current density–voltage (*J*–*V*) curves in forward and reverse direction of the champion devices. e) The summery of the device efficiencies for ion‐free Spiro‐based HTLs.

According to these results, a working mechanism of charge transport in Spiro‐based HTLs was proposed as shown in **Figure** **2**a. For devices doped with Li + TBP, enhancing the concentration of TFSI^−^ anions improves carrier mobility by reducing energy barriers for hole hopping.^[^ ^45^ ^]^ For the F4TCNQ‐only‐doped devices, although a large number of free carriers was generated due to the superior electron‐accepting capacity of the F4TCNQ, its low carrier mobility leads to high‐energy barrier for hole hopping and poor charge‐transport capacity. In comparison, the intramolecular charge transport through P3HT chains in the film of Spiro + F4TCNQ + P3HT provides additional transport pathways for the charge carriers, which increase the film conductivity. In addition, P3HT provides a high HOMO energy level to well match with the LUMO of F4TCNQ, which favors interfacial hole transfer. Overall, the synergistic effects of F4TCNQ and P3HT achieve a superior mobility and conductivity in the ion‐free Spiro‐based HTLs.

We prepared PSCs with an ITO/SnO_2_/perovskite (PSK)/Spiro/Ag device structure (Figure 2b) to investigate the doping effect on the photovoltaic performance of the devices. Figure 2c and S4, Supporting Information, show the statistical distribution of PCEs for different devices. It is clear that the PCEs increased significantly after adding either P3HT or F4TCNQ compared to those with undoped Spiro. The improved PCEs are mainly ascribed to the increased fill factor (FF) and short‐circuit current density (*J*_sc_) (Figure S5, Supporting Information), which could be mostly attributed to the improved hole mobility in the HTLs. However, the devices exhibited relatively low open‐circuit voltage (*V*_oc_), which could be related to the poor physical contact between the perovskite and macromolecule P3HT.^[^ ^46^ ^]^ We also examined the performance of the devices with bare P3HT as HTL, as shown in Figure S6, Supporting Information. The results further indicate that inferior interfacial properties of P3HT limit the device efficiency. As the ratio of P3HT increases, P3HT started to aggregate in the Spiro film (Figure S7, Supporting Information), which led to a sharp decrease of PCEs. As the content of F4TCNQ increases, the devices showed lower PCEs which could be the result of the accumulated abundance of photogenerated carriers at the interface between perovskite and HTL, leading to high non‐radiative recombination losses. We further optimize the mixing ratio between Spiro and P3HT by tuning their volume ratio from 99.5:0.5, 99:1, 97.5:2.5 to 95:5 based on the same stock solution (Spiro solution:72 mg mL^−1^; P3HT solution:10 mg mL^−1^). As shown in Figure S8, Supporting Information, an optimal volume ratio 99:1 has been obtained to achieve the highest performance. The optimal *J–V* curves and solar photovoltaic parameters of the PSCs are shown in Figure 2d and Table S1, Supporting Information. The device with Spiro + P3HT showed the best PCE of 17.53% with a *V*_oc_ of 0.97 V, a *J*_sc_ of 23.62 mA cm^−2^, and an FF of 76.76%. The device based on Spiro + F4TCNQ showed the best PCE of 19.07% with a *V*_oc_ of 1.05 V, a *J*_sc_ of 23.00 mA cm^−2^, and an FF of 78.67%. Both devices achieved higher PCEs than the device with undoped Spiro (PCE = 14.97%, *J*_sc_ = 22.83 mA cm^−2^, *V*_oc_ = 1.02 V, and FF = 64.09%). Encouragingly, the CTC‐based HTLs further promoted the device efficiency to 20.14% (*J*_sc_ = 23.71 mA cm^−2^, *V*_oc_ = 1.07 V, and FF = 78.96%), which represent the highest reported PCEs so far for all the PSCs based on ion‐free Spiro‐based HTLs, as summarized in Figure 2e and Table S4, Supporting Information. We also observed strong light soaking effect for the molecule‐doped devices. Figure S9, Supporting Information, shows the increase of the device performance based on Spiro + F4TCNQ + P3HT during light soaking. The *J*_sc_ was increased from 23.05 to 23.61 mA cm^−2^, and the light soaking effect is quite reversible during dark–light cycles. The external quantum efficiency (EQE) spectra of the representative Spiro + F4TCNQ + P3HT devices were characterized to evaluate the *J*_sc_. The integrated current from EQE is 21.93 mA cm^−2^, which is over 95% of initial *J*_sc_ derived from the *J–V* scans, as shown in the Figure S10, Supporting Information. As shown in Table S2, Supporting Information, the hysteresis index (HI) of Spiro + F4TCNQ + P3HT device (HI = 0.1) is lower than Spiro (HI = 0.13). CTC‐based devices produce more carrier concentration and provide multiple carrier‐transport channels, which decreases the charge accumulation at the perovskite interfaces and favors decreasing the hysteresis phenomenon, as discussed in previous report.^[^ ^47^ ^]^ Overall, the CTC‐based device exhibited the better performance among all molecular‐doped HTL. However, the PCEs of the molecular‐doped devices were generally lower than the ion‐doped devices. For ionic dopants, a large number of anions are introduced to improve the carrier mobility because the interaction between anions and the holes could reduce the energy barriers for the hole hopping. In contrast, the lack of the anions in the case of molecular dopants limits the carrier transportation and extraction.

**Figure 3:**
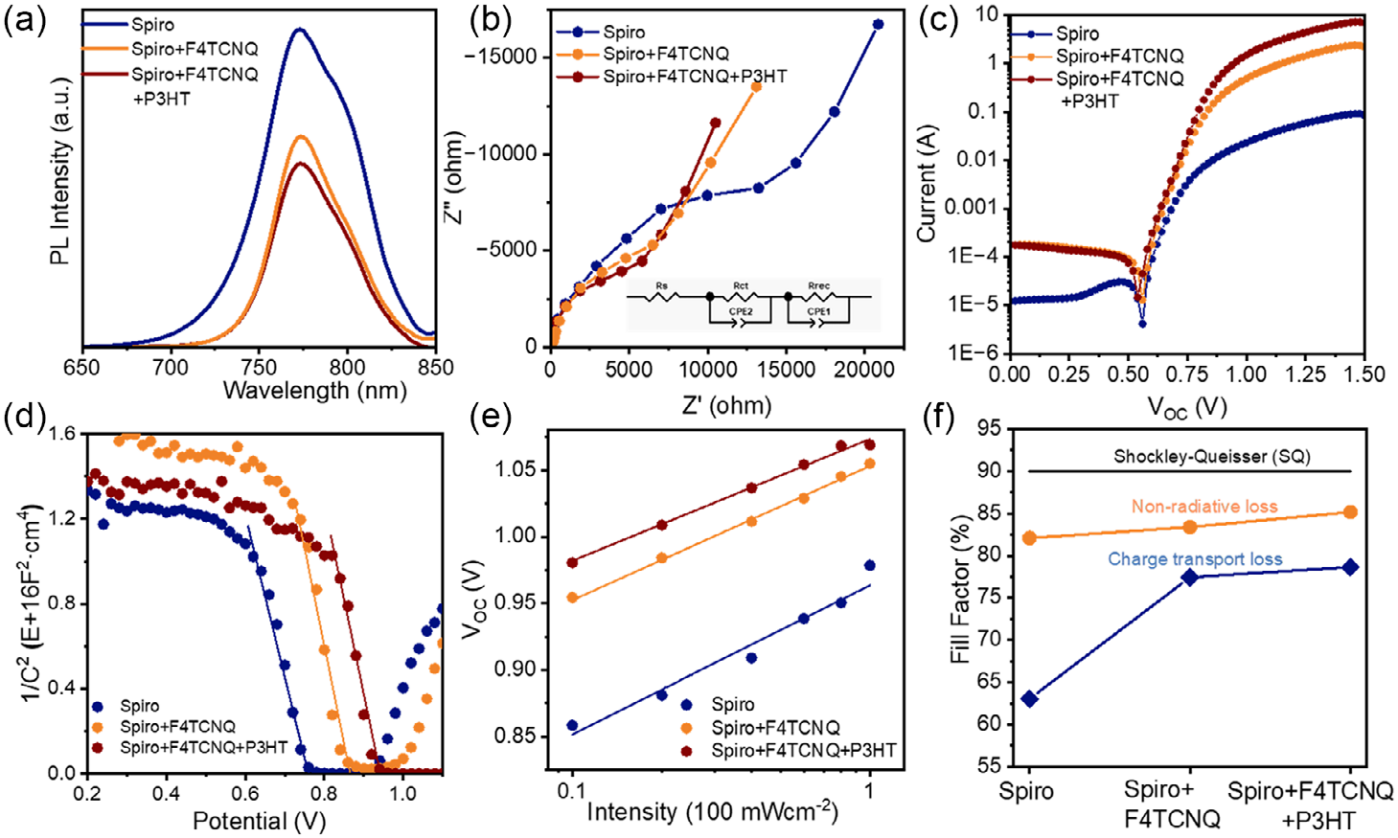
a) Photoluminescence spectra of samples based on different molecular additives (perovskite/HTLs). b) Electrochemical impedance spectroscopy, c) dark current, d) Mott–Schottky measurement, e) light‐dependent open‐circuit voltage (*V*_oc_) measurements, and f) the limiting factors of fill factor.

The interfacial hole‐transfer efficiency was monitored for different samples by steady‐state photoluminescence to further investigate the influence of different additives on the charge‐transport kinetics at the perovskite/HTL interface. **Figure** **3**a displayed that the luminescence quenching of the Spiro + F4TCNQ + P3HT‐based device was significantly increased compared to that of the Spiro and Spiro + F4TCNQ devices, indicating a superior hole‐extraction ability to reduce the carrier accumulation at the interfaces.^[^ ^48^ ^]^ To further compare the interfacial resistance in different devices, electrochemical impedance spectroscopy was applied (Figure 3b). Table S3, Supporting Information, summarizes the *R*_CT_, which reflects the charge‐transfer barrier at the perovskite/Spiro/Ag interface.^[^ ^49^ ^]^ It can be seen that the Spiro + F4TCNQ + P3HT devices showed much lower resistance than the others. Figure 3c shows the dark current (*J*_dark_) curves based on different devices under dark conditions. From the plot of *J*_dark_, it can be concluded that the improvement of FF is most likely due to the lower series resistance in the Spiro films.^[^ ^50^ ^]^ Mott–Schottky measurements were also performed in darkness to reflect the driving power for interfacial charge extraction, capacitance–voltage (*C–V*) curves are shown in Figure 3d. The built‐in potential (*V*_bi_) of Spiro + F4TCNQ + P3HT‐based device was estimated to be 0.94 V, which is higher than *V*_bi_ of Spiro + F4TCNQ (0.87 V) and Spiro (0.77 V). This further implies a greater carrier extraction capability at the perovskite interfaces.^[^ ^51^ ^]^ Light‐dependent *V*_oc_ measurements were performed to better explore the bulk and interfacial carrier recombination as shown in Figure 3e. The slope of the fitted lines can be used to estimate the ideality factor (*n*_id_), which could be exploited to identify the different charge recombination processes. Since all the devices were applied with the same n–i–p structure except different additives in the HTLs, the reduction of *n*_*i*d_ indicates that the trap‐assisted carrier recombination at the perovskite‐HTL interface was efficiently inhibited after the addition of both F4TCNQ and P3HT. Furthermore, another approach to analyze the FF loss based on different additives was provided by the theoretical values calculated from *n*_id_ with the measured values. As shown in Figure 3f, the Spiro device showed considerable charge‐transport losses, which appeared to limit the FF. In contrast, the device with CTC exhibited reduced charge‐transport losses, thereby improving the photovoltaic parameters.

**Figure 4:**
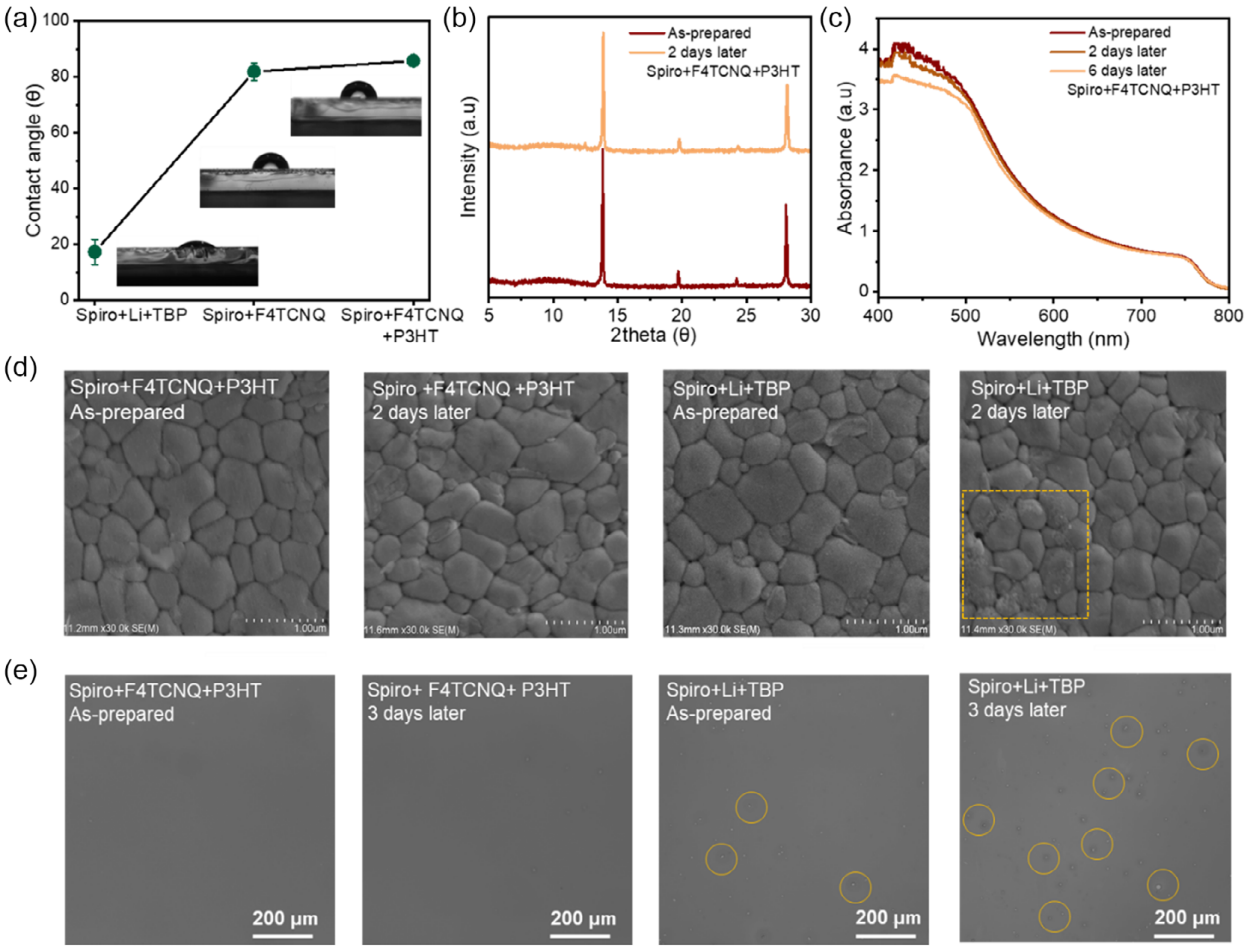
a) The water contact angles (*θ*) on Spiro films doped with various additives. b) X‐ray diffraction patterns of as‐prepared films (ETL/PSK/HTL) and the devices after 2 days in outdoors environment (25 °C, 65%RH) based on Spiro + F4TCNQ + P3HT. c) UV–vis of as‐prepared films (ETL/PSK/HTL) in outdoor environment (25 °C, 65%RH) based on Spiro + F4TCNQ + P3HT. d) Top‐view scanning electron microscopic (SEM) images of the perovskite films placed in outdoor environment (65%RH, 25 °C). e) Polarizing microscope images of the Spiro films placed under continuous light‐emitting diode illumination (35%RH).

To study the effect of molecular doping on the hydrophobicity of HTLs, we obtained the different contact angles (*θ*) of water for the Spiro samples with various dopants. The contact angle values of the Spiro films were measured using deionized water to compare their hydrophobic properties (**Figure** **4**a). Encouragingly, the films with Spiro + P3HT (81.9°) and Spiro + F4TCNQ + P3HT (85.7°) showed significantly higher hydrophobicity than the film with Spiro + Li + TBP (17.3°), which would be favorable for protecting the perovskite film from the environment. To prove this, we placed the HTL‐coated perovskite samples in a humid outdoor environment (65% relative humidity (RH), 25 °C). As seen in the X‐ray diffraction (XRD) in Figure 4b and S11a, Supporting Information, the sample with Spiro + Li + TBP developed a significant peak at 2*θ* = 12.5°, attributed to PbI_2_, after 2 days of ambient storage. In contrast, negligible change in XRD spectra was observed for the film with Spiro + F4TCNQ + P3HT, which confirms the positive role of CTC in enhancing the stability of the perovskite film compared to the one with ionic dopants.

**Figure 5:**
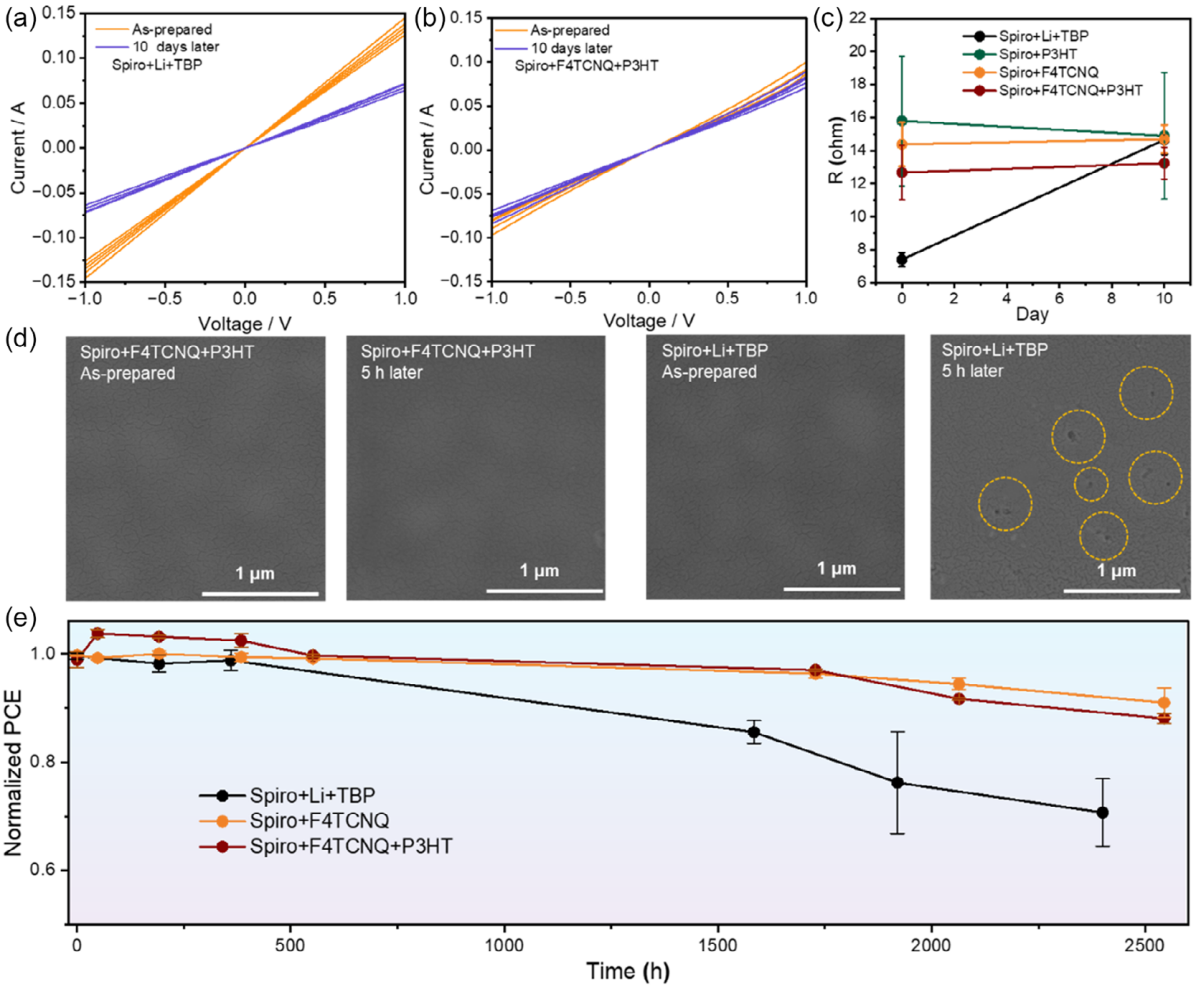
The change of *I–V* curves (five devices were measured for each condition) for the Spiro films with a) Li + TBP and b) F4TCNQ + P3HT. c) The change of resistivity for the Spiro samples with different additives for 10 days. d) SEM images of the Spiro films exposed at 85 °C in indoor environment (35%RH). e) Long‐term environmental stability of devices (25 °C, 25%RH) for 2500 h.

This was further confirmed by the little change in the light absorption of the film with Spiro + F4TCNQ + P3HT (Figure 4c and S11b, Supporting Information). Figure 4d shows the SEM images of the perovskite films coated with Spiro films based on different dopants after 2 days of outdoor storage (note: the Spiro film was washed off before the test). The perovskite film based on Spiro + Li + TBP degraded severely into small particles (see the area in yellow frame), which could be mostly caused by the high hygroscopicity of LiTFSI.^[^ ^52^ ^]^ While CTC‐based perovskite film maintained complete grains without obvious degradation. The CTC‐based films also show improved light stability. As shown in Figure 4e, the film with Spiro + F4TCNQ + P3HT exhibited negligible morphological change under LED illumination for 3 days, whereas the film based on Spiro + Li + TBP showed notable white‐dots defects across the film under the same condition. This feature was mostly resulted from the LiTFSI aggregation after illumination.^[^ ^53^ ^]^ We also observed grey agglomeration and high roughness in the Ag‐electrode area after 10 days of illumination, which could be related to the Li^+^ diffusion and the hygroscopicity of LiTFSI (Figure S12a, Supporting Information). On the contrary, the Ag layer deposited on the CTC‐based film showed much more intact and smoother morphology. We also monitored the change of the HTL resistivity under continuous illumination. Little change of the film resistivity for molecular‐doped Spiro films further proves the enhanced film stability compared to ion‐doped films (**Figure** **5**a–c and S12b,c, Supporting Information).

We also evaluated the morphological change of the Spiro layer with different dopants under 85 °C for 5 h in the ambient condition (Figure 5d). In contrast to large number of pinholes formed in the Spiro + Li + TBP film, few pinholes were appeared in the CTC‐based film. We finally tested the device stability under different conditions. The devices were stored in the drying cabinet (RH = 25%) to evaluate their shelf‐lifetime. The devices based on Spiro + F4TCNQ and Spiro + F4TCNQ + P3HT retained approximately 90% of the initial efficiency after 2500 h (Figure 5e), which showed much higher stability than those based on Spiro + Li + TBP (70%) under the same conditions. The maximum power point stability was characterized for the devices without any encapsulation under ambient conditions for 25 h in Figure S13, Supporting Information. The CTC‐based device demonstrated outstanding stability by maintaining 97% of the initial PCE after about 25 h, while the device with Spiro + Li + TBP underwent dramatic degradation after 5.5 h. Figure S14, Supporting Information, shows the humid stability of different devices under a condition of 75% RH. After about 10 days, the efficiency of the Spiro + F4TCNQ + P3HT devices remained 90% of the initial PCEs, showing much higher stability than the devices based on Spiro + Li + TBP. These results all indicate that the Spiro + F4TCNQ + P3HT components show high promises in film and device stability compared to the Spiro + Li + TBP components.

## 3 Conclusion

The stability issues of the ion‐doped HTL have been the major factor limiting the reliability and application of PSCs. In this work, we developed a strategy of CTC by combining multifunctional molecules in the HTL. The CTC strategy suppressed the phase segregation and thus improved the HTL stability under the conditions of heat, light, and humid conditions. We also analyze the individual and collective effects of the molecular dopants on charge‐transport and interfacial properties in the devices. We find that the addition of F4TCNQ is critical for doping both Spiro and P3HT, and the key role of P3HT is to provide additional charge‐transport channels for increasing the carrier mobility. As a result, the devices based on CTC achieved a notable increase of PCE of up to 20.14% in comparison to14.97% for the devices without molecular additives, which represents the highest reported efficiency for ion‐free Spiro‐based HTLs in PSCs. More encouragingly, the CTC‐based devices without any encapsulation demonstrated remarkable environmental stability, maintaining 90% of the initial efficiency after about 2500 h under ambient conditions. This work provides new insights into understanding the working mechanism of molecular additives, and gives new inspiration for further improving the PSCs performance.

## Conflict of Interest

The authors declare no conflict of interest.

## Supporting information

Supplementary Material

## Data Availability

The data that support the findings of this study are available from the corresponding author upon reasonable request.
