# Supplementary material for: Molecular Cooperation of Ion‐Free Ternary Complexes Enhances Efficiency and Stability of Perovskite Solar Cells
Source: Small Sci. 2023 Nov 27;4(1):2300165. doi: 10.1002/smsc.202300165 (PMC11935208; doi:10.1002/smsc.202300165)
Supplement: Supplementary file 1 — Supplementary Material [file SMSC-4-2300165-s001.pdf]

Supporting Information

Materials:

Isopropyl Alcohol (IPA) and Acetonitrile (ACN) were purchased form Energy Chemical. Ethyl acetate (EA), 4-tert-butyl pyridine (TBP), Dimethyl sulfoxide (DMSO), Chlorobenzene (CB) and N, N-Dimethylformamide (DMF) were obtained from Sigma-Aldrich. Tin (iv) oxide colloid precursor (SnO_2, 15% in H_2O colloidal dispersion) was purchased from Alfa Aesar. 2, 2', 7, 7'-tetrakis (N, N-di-p-methoxy-phenylamine)-9, 9'-spirobifuorene (spiro-OMeTAD), Methylamine chloride (MACl), Methylammonium iodide (MAI), Formamidinium Bromide (FABr), Cesium Iodide (CsI) and Indium Tin Oxide film (ITO) were purchased from Advanced Election Technology Co.,LtD. Iso-Butylammonium BromideSynonium (i-BABr), Lithium bis (triuoromethanesulfonyl) imide (LiTFSI), Lead (II) bromide ($PbBr_2$) and Poly(3-hexylthiophene-2,5-diyl) (P3HT) were obtained from Xi'an Polymer Light Technology Crop. 2,3,5,6-Tetrafluoro-7,7,8,8-tetracyanoquinodimethane (F4TCNQ) was purchased from Macklin. Lead (II) iodide (PbI_2) was purchased from TCI. All materials mentioned above were used as received without further purification. Formamidine iodide (FAI) was synthesized in own laboratory.

Preparation of hole transport materials (HTMs) solution:

The Spiro solution was prepared by dissolving 72.3 mg Spiro-OMeTAD in 1 mL CB without any additives.

The Spiro with Li-TBP solution was prepared by dissolving 72.3 mg Spiro-OMeTAD, 28.8 μL TBP and 17.5 μL LiTFSI (520 mg LiTFSI in 1 mL ACN) in 1 mL CB.

The Spiro solution was prepared by dissolving 72.3 mg Spiro-OMeTAD in 1 mL CB. And the P3HT solution was prepared by dissolving 10 mg P3HT in 1 mL CB. The Spiro+P3HT solution was prepared by mixing the aforementioned Spiro solution and P3HT solution together with different volume ratios, for example, volume (Spiro

solution):volume (P3HT solution)=99:1, 97.5:2.5 and 95:5.

The Spiro with F4TCNQ solution was prepared by dissolving 72.3 mg Spiro-OMeTAD and 0.5 mg, 1 mg or 1.5 mg F4TCNQ in 1 mL CB.

The Spiro with F4TCNQ solution was prepared by dissolving 72.3 mg Spiro-OMeTAD in 1 mL F4TCNQ solution (0.5 mg/mL F4TCNQ in CB). The P3HT solution was prepared by dissolving 10 mg P3HT in 1 mL CB. The Spiro+F4TCNQ+P3HT solution was prepared by mixing the aforementioned Spiro with F4TCNQ solution and P3HT solution together with different volume ratios, for example, volume (Spiro with F4TCNQ solution):volume (P3HT solution)=99.5:0.5, 99:1, 97.5:2.5 and 95:5.

Device fabrication:

The ITO was sliced into 2 × 2 cm^2 substrate. The ITO substrate was cleaned with detergent, glass cleaning fluid (PV-tech, consisting of surfactant and dispersant), deionized water (DI), acetone and IPA for 10 min each. The dry ITO was treated with UV-ozone for 20 min to improve wettability. Under air condition, SnO_2 film (nanoparticle solution mixed with DI in volume ratio of 1:3) was spin-coated on the surface of ITO at 2500 rpm for 30 s and annealed at 200℃ for 40 min. The wetting surface of the electron transport layer (ETL) was enhanced by UV-ozone for 15 min. Then, 40 μL $FA_{0.9}Cs_{0.07}MA_{0.03}Pb (I_{0.92}Br0._{08})_3$ perovskite precursor solution (79.4 mg $PbBr_2$, 27.3 mg FABr, 380.6 mg FAI, 63.5 mg MACl, 12.6 mg MAI and 1182.9 mg PbI_2, 49.1 mg CsI were dissolved in 400 mL DMF and 1602 mL DMSO) was spin-coated on the substrate at 1000 rpm for 10s and 4000 rpm for 30s. In the second procedure, 260 μL EA was dropped on the substrate for the last 10 s and the film was annealed at 100 °C for 40 min in air condition. The passivation layer was prepared by dynamically spin-coating i-BABr solution (2.3 mg in 1 mL IPA) on the perovskite layer and annealing at 100 °C for 5 min in a glove box. The prepared HTMs solution was spin-coated on the substrate at 4500 rpm for 30 s to fabricate the hole transport layer. Finally, the device was completed by evaporating 80 nm Ag contact electrode under a vacuum level of 3.5×10^{-3} Pa through a 0.09 cm^2 mask.

Film and device characterizations:

The *J-V* curves were recorded using a Keithley 2400 source meter. A solar simulator (SS-X50, ENLITECH, Taiwan China, 3A class) with a xenon light source (LSB-X150A0FR, ZOLIX) was used to generate the AM 1.5G irradiance (100 mW/cm^2). The simulated solar illumination was controlled by a calibrated crystalline Si cell. Scanning ranges from 1.2 V to -0.1 V and -0.1 V to 1.2 V with a 0.04 cm^2 mask. Top view scanning electron microscopy (SEM) images were characterized using an SU-70 scanning electron microscope. Fourier transform infrared (FTIR) spectroscopy of Spiro-OMeTAD films deposited on ITO was performed in the Nicolet is. X-ray diffraction patterns were measured using a Bruker-axs XRD (D8-A25). The contact angle was measured using a video-optical contact angle meter (DSA20). Ultraviolet-visible (UV-Vis) absorption spectra were obtained using a UV-Vis spectrophotometer (Shimadzu UV-2550). The capacitance-voltage (C-V) and electrochemical impedance spectra (EIS) of the finished devices, mobility and resistivity of the films were measured using an electrochemical workstation (CHI600E). Photoluminescence (PL) spectra were recorded with a transient fluorescence spectrometer (Edinburgh FLS1000) using 450 nm light for steady state excitation. Polarizing microscope images were obtained using a DM600B polarizing and fluorescence microscope (Leica).

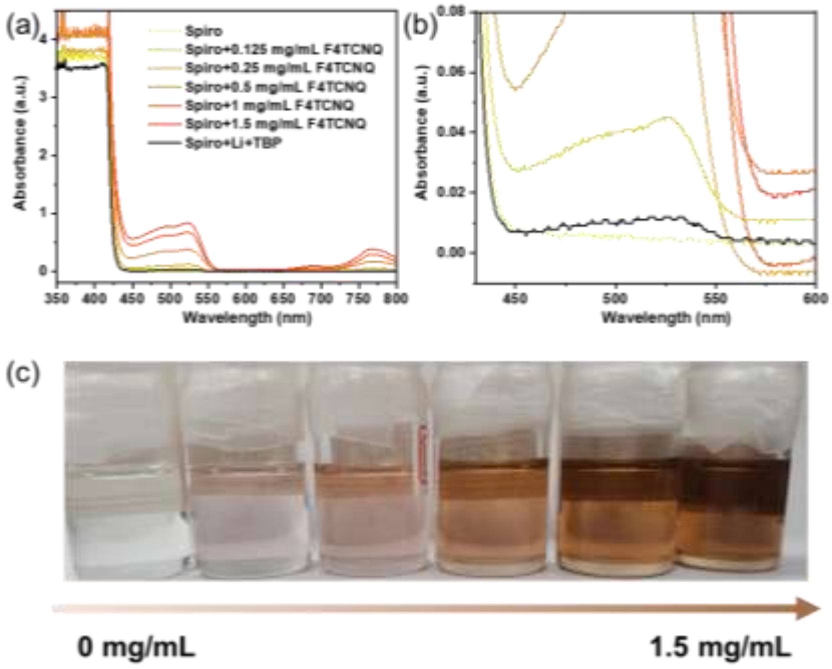

Figure S1. (a, b) UV-vis absorption spectra of Spiro solution doped by F4TCNQ with different concentration and Spiro+Li+TBP. (c) Digital photographs of Spiro solution doped by F4TCNQ with different concentration.

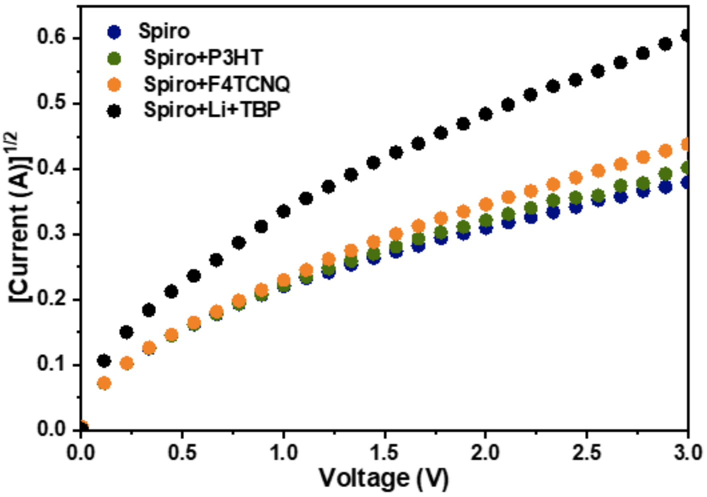

Figure S2. Hole mobility tests of spiro films with different additives.

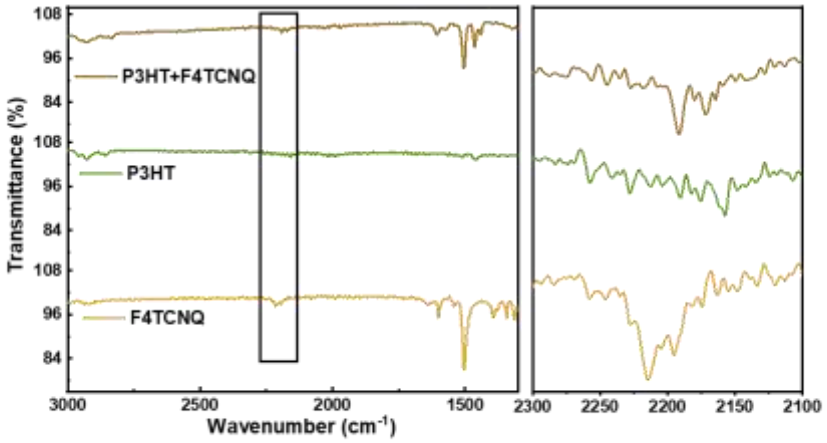

Figure S3. FTIR spectra of P3HT, F4TCNQ and P3HT+F4TCNQ films.

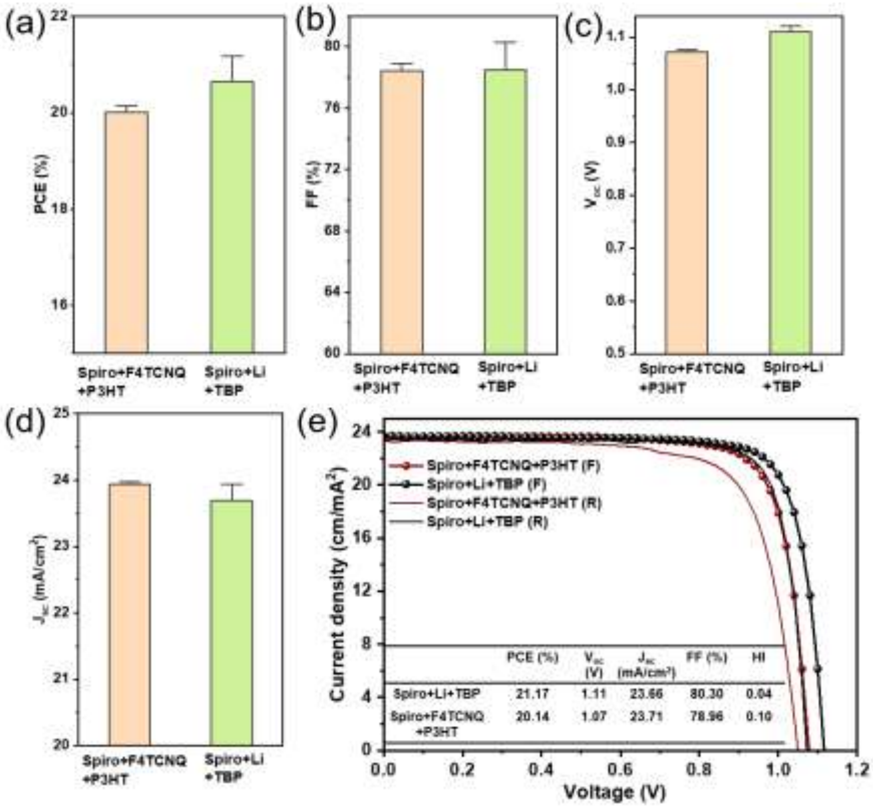

Figure S4. The statistical distribution in forward direction of (a) PCE, (b) V_{oc}, (c) *FF* and (d) J_{sc} for different devices. (e) Current density (*J*)- voltage (*V*) curves of champion devices and the photovoltaic parameters in forward direction inset.

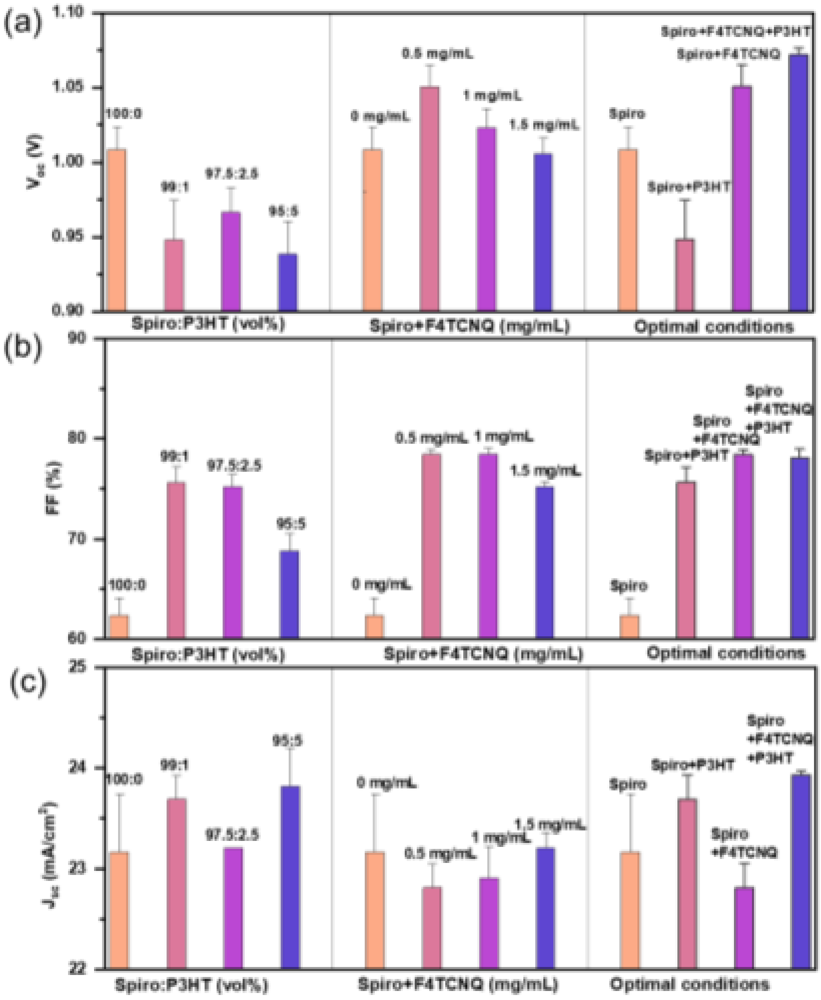

Figure S5. The statistical distribution of V_{oc}, *FF* and J_{sc} in forward direction for different devices.

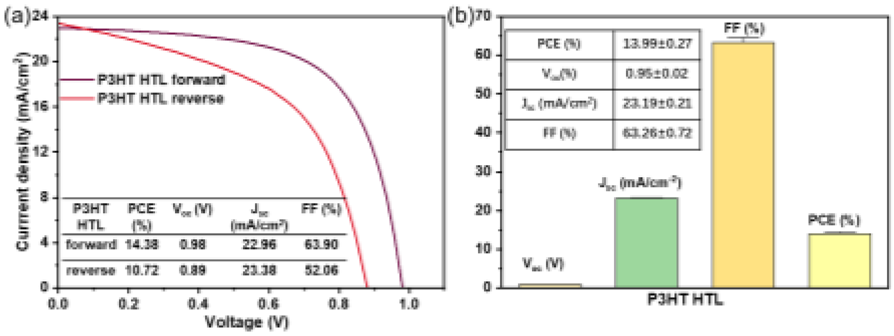

Figure S6. (a) Current density (*J*)- voltage (*V*) curves of P3HT based devices and the photovoltaic parameters inset. (b) The statistical distribution in forward direction of V_{oc}, *FF* and J_{sc} for P3HT based devices.

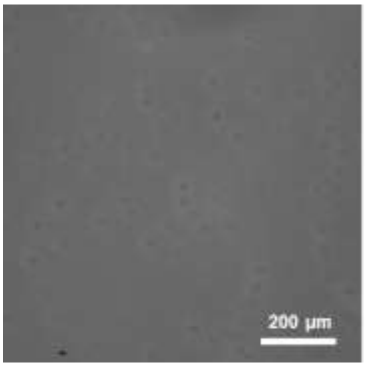

Figure S7. Polarizing microscope images of the films based on Spiro+P3HT (ratio of 95:5).

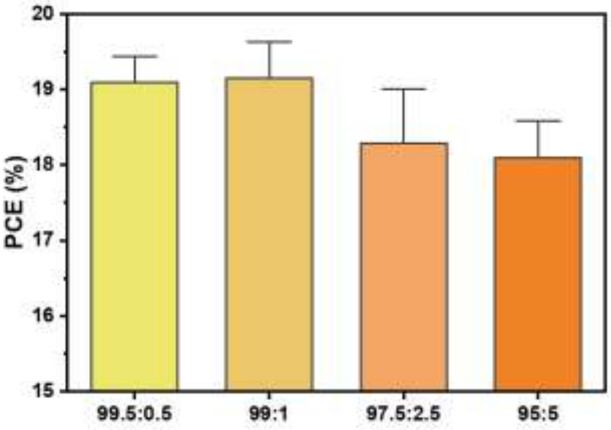

Spiro+F4TCNQ +P3HT	PCE (%)	V_{oc} (V)	J_{sc} (mA/cm^2)	FF (%)
99.5:0.5	19.09±0.36	1.04 ±0.01	23.27 ±0.30	79.04±0.91
99:1	19.15 ±0.36	1.05 ±0.01	23.16 ±0.32	78.84 ±1.79
97.5:2.5	18.29 ±0.59	1.03 ±0.01	23.09 ±0.37	76.56 ±2.44
95:5	18.10 ±0.50	1.03 ±0.01	23.03 ±0.26	76.41 ±2.01

Figure S8. The statistical distribution in forward direction of V_{oc}, *FF* and J_{sc} for Spiro+F4TCNQ+P3HT based devices (0.5 mg/mL F4TCNQ; Spiro: P3HT volume ratio: 99.5:0.5, 99:1, 97.5:2.5 and 95:5).

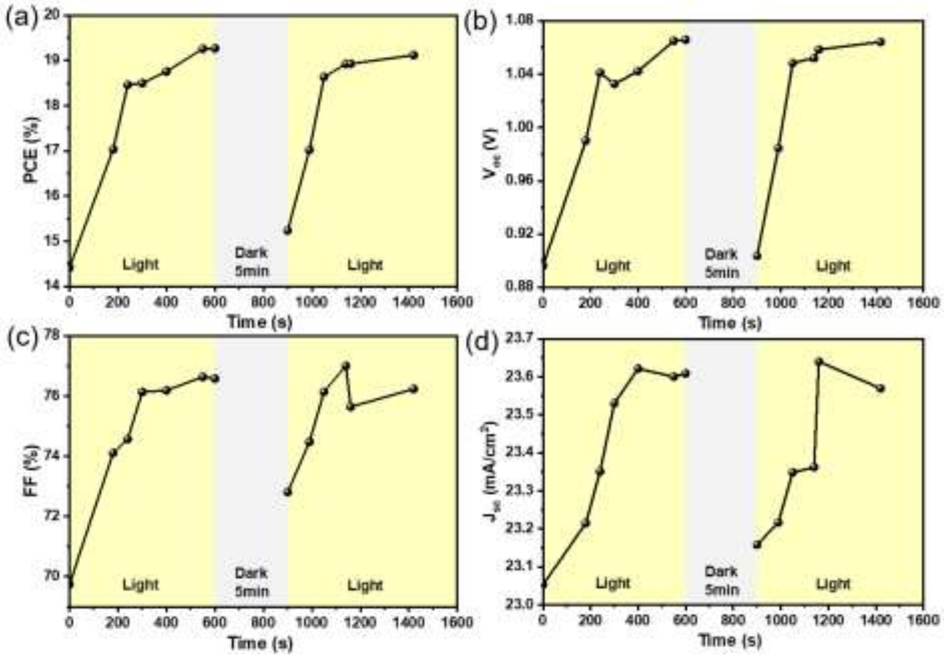

Figure S9. Evolution of (a) PCE, (b) V_{oc}, (c) FF and (d) J_{sc} during light exposure.

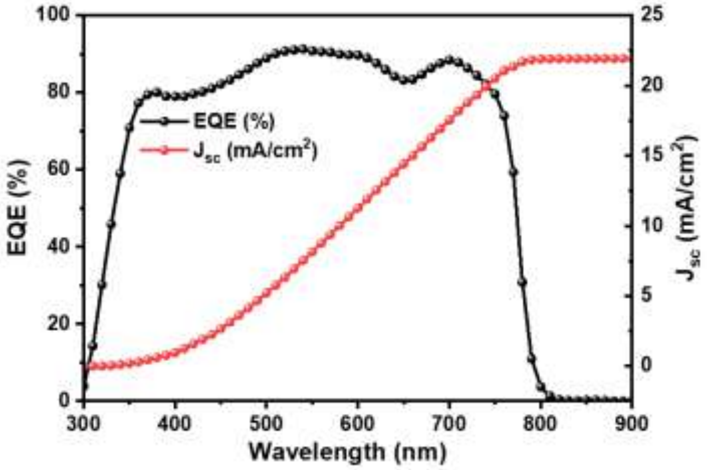

Figure S10. EQE of the devices based on Spiro+F4TCNQ+P3HT.

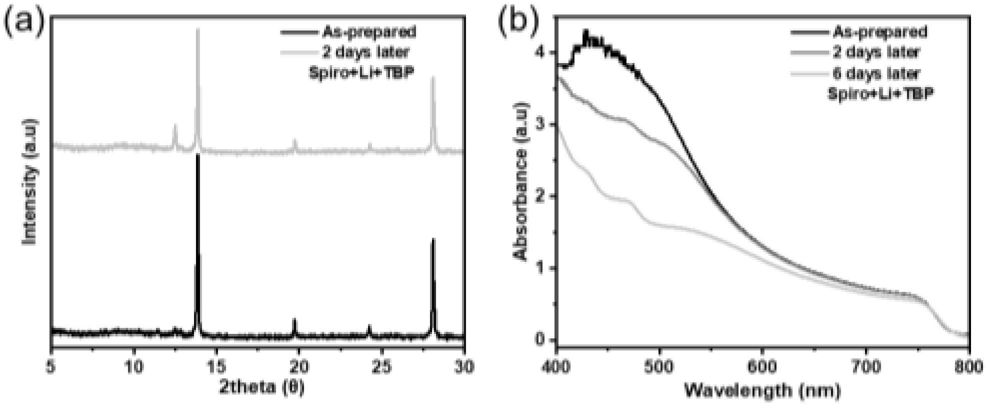

Figure S11. (a) XRD patterns of as-prepared films (ETL/PSK/HTL) and the devices after 2 days in outdoors environment (25°C, 65%RH) based on Spiro+Li+TBP. (c) UV-vis of as-prepared films (ETL/PSK/HTL) in outdoor environment (25°C, 65%RH) based on Spiro+Li+TBP.

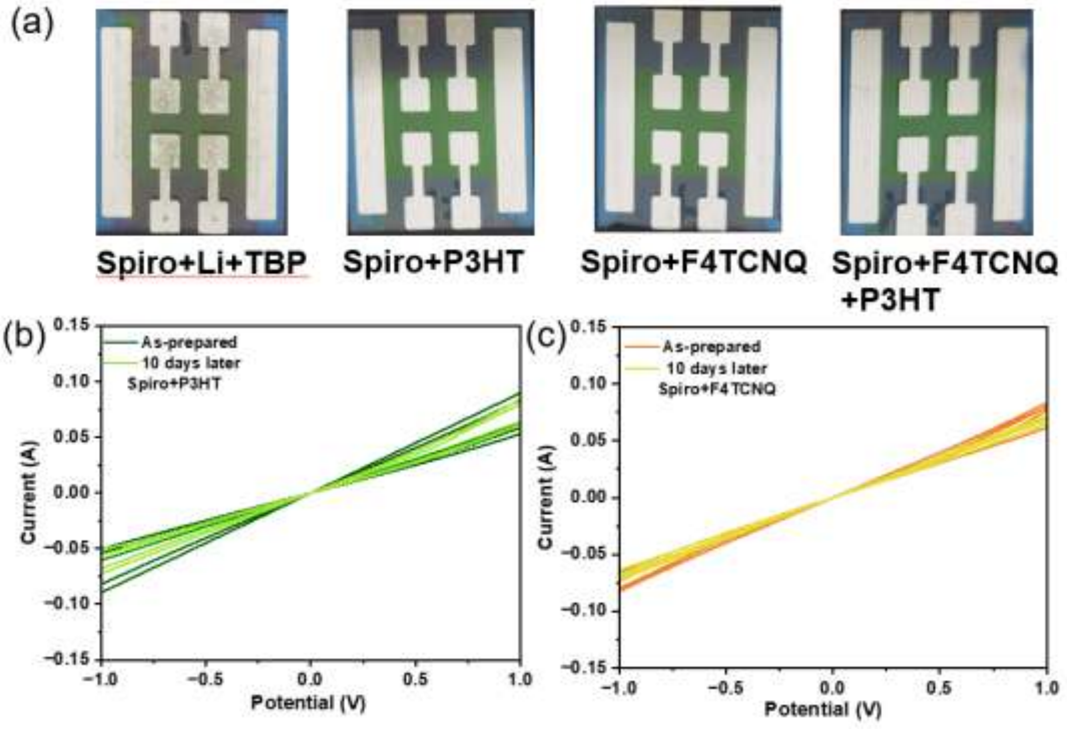

Figure S12. (a) Digital photographs of Spiro films with different additives after 10 days illumination. The change of *I-V* curves for the spiro films with P3HT (b), F4TCNQ (c) during 10 days illumination.

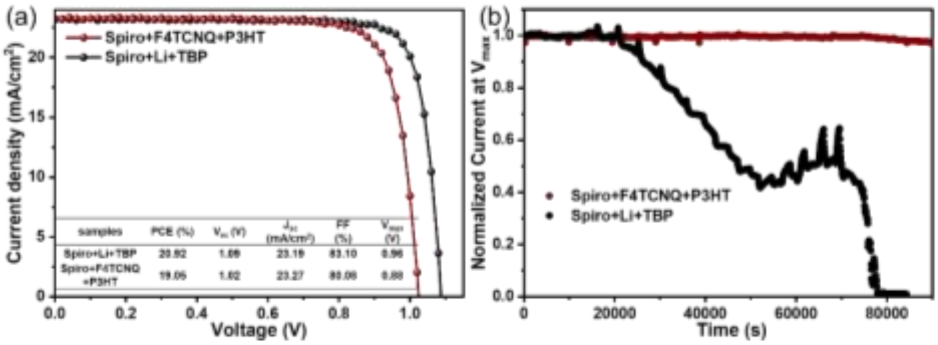

Figure S13. (a) Initial current density (*J*)- voltage (*V*) curves in forward direction of devices. (b) The evolution of current of the devices without any encapsulation under ambient conditions for 25 h.

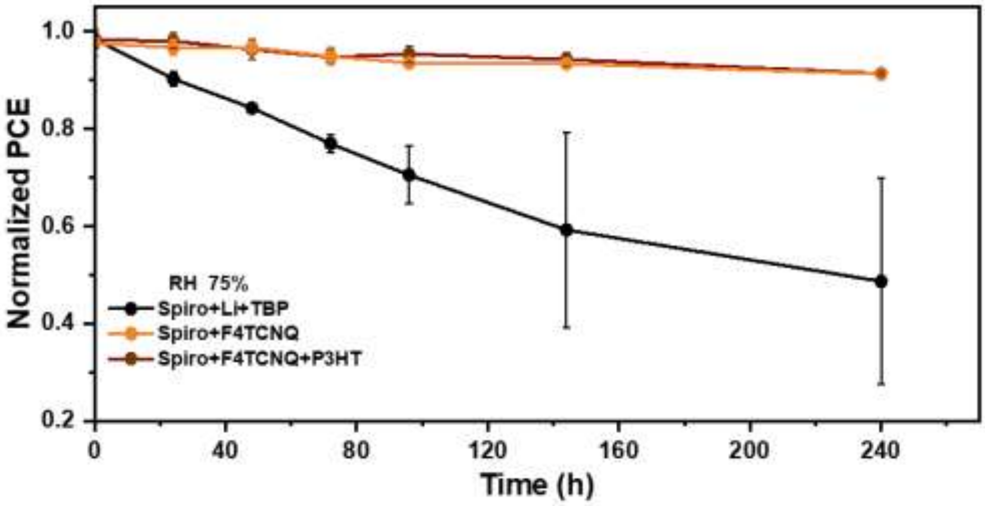

Figure S14. Humid stability of devices under 75% RH for 10 days.

	PCE (%)	V_{oc} (V)	J_{sc} (mA/cm^2)	FF (%)
Spiro: P3HT=99:1	17.00±0.50	0.95±0.03	23.69±0.12	75.62±1.52
Spiro: P3HT=97.5:2.5	16.87±0.21	0.97±0.01	23.21±0.19	75.18±1.16
Spiro: P3HT=95:5	15.38±0.42	0.94±0.01	23.82±0.37	68.82±1.62

	PCE (%)	V_{oc} (V)	J_{sc} (mA/cm^2)	FF (%)
Spiro+0.5 mg/mL F4TCNQ	18.79±0.19	1.05±0.01	22.82±0.23	78.42±0.46
Spiro+1 mg/mL F4TCNQ	18.38±0.27	1.02±0.01	23.00±0.33	78.44±0.53
Spiro+1.5 mg/mL F4TCNQ	17.54±0.20	1.01±0.01	23.21±0.14	75.18±0.41

	PCE (%)	V_{oc} (V)	J_{sc} (mA/cm^2)	FF (%)
Spiro	14.56±0.27	1.01±0.01	23.17±0.38	62.34±1.15
Spiro+F4TCNQ+P3HT	20.02±0.09	1.07±0.01	23.83±0.14	78.03±0.55
Spiro+Li+TBP	20.65±0.44	1.11±0.01	23.69±0.14	78.47±1.36

Table S1. The PCE, V_{oc}, J_{sc} and *FF* in forward direction of the cells based on different additives.

	PCE (%)	V_{oc} (V)	J_{sc} (mA/cm^2)	FF (%)	HI
Spiro	14.97	1.02	22.83	64.09	0.13
Spiro+P3HT	17.53	0.97	23.62	76.76	0.12
Spiro+F4TCNQ	19.07	1.05	23.00	78.67	0.11
Spiro+F4TCNQ+P3HT	20.14	1.07	23.71	78.96	0.10

Table S2. The PCE, V_{oc}, J_{sc}, *FF* in forward direction and HI of the champion cells based on different molecular additives.

samples	R_{CT} (ohm)	V_{bi} (V)	n_{id}
Spiro	21104	0.77	1.73
Spiro+F4TCNQ	13966	0.87	1.67
Spiro+F4TCNQ +P3HT	9059	0.94	1.46

Table S3. The R_{CT}, V_{bi} and n_{id} of cells based on different molecular additives.

Table S4. Device efficiency from literature based on ion-free Spiro-OMeTAD

Ion-free spiro in perovskite solar cell						
Year	PCE (%)	Device structure	Method	Perovskite components	pixel area	
2023	10.45	FTO/SnO_2/TiO_2/Perovskite/CNTs-Spiro-OMeTAD /Carbon	Carbon nanotubes (CNTs)+Spiro-OMeTAD	$MAPbI_3$		1
2022	20.02	ITO/SnO_2//Perovskite/Spiro-OMeTAD/Au	Evaporated Undoped Spiro-OMeTAD	$FA_{0.90}Cs_{0.07}MA_{0.}$ $_{2.7}6Br_{0.24}$	0.04 cm^2	2
2022	14.6	FTO/c-TiO_2/m-TiO_2/ Perovskite/ Spiro-OMeTAD /Au	Spiro-OMeTAD+F4TCNQ	Cs0.05FA0.81MA0.14 PbI2.55Br0.45	20 mm^2	3
2021	10.95	FTO/TiO_2/Perovskite/NiO/ Spiro-OMeTAD	Spiro-OMeTAD+NiO	$MAPbI_3$		4
2020	20.13	ITO/SnO_2/Perovskite/Spiro-OMeTAD /MoO_3/Ag	Spiro-OMeTAD+ Tris(pentafluorophenyl)borane (TPFB)	$Cs_{0.05}FA_{0.80}MA_{0.15}PbI_{2.5}$ $_{5}Br_{0.45}$		5
2020	11.75	FTO/c-TiO_2/m-TiO_2/Perovskite/Spiro-OMeTAD/Au	Spiro-OMeTAD+F4TCNQ	$MAPbI_3$		6
2019	17	ITO/C60/Perovskite/Spiro/MoO_3/Ag	Dopant-free Spiro-OMeTAD dissolved in Tetrahydrofuran	MAPbI3-xClx	0.04 cm^2	7
2019	16.92	ITO/SnO_2/Perovskite/Spiro-OMeTAD /Au	Optimizing the thickness of dopant-free Spiro-OMeTAD layer (50nm)	$MAPbI_3$	0.09 cm^2	8
2019	18.7	ITO/SnO_2//Perovskite/Spiro-OMeTAD/Au	Spiro-OMeTAD+Water free PEDOT	$Cs_{0.05}((CH(NH_2)_2)_{0.83}(CH3NH_3)_{0.17})_{0.95}Pb(I_{0.83}Br_{0.17})_3$	0.16 cm^2	9
2018	18.01	FTO/TiO_2/Perovskite/Spiro-OMeTAD/Au	Perovskite+Benzoquinone (BQ) and Spiro-OMeTAD+F4TCNQ	$MAPbI_3$	0.066 cm^2	10
2017	12.93	FTO/compact TiO_2/Perovskite/Spiro-OMeTAD /Au	Spiro-OMeTAD+F4TCNQ	$CH_3NH_3PbI_3$	0.15 cm^2	11
2016	18.02	FTO/TiOx/Perovskite/Spiro-OMeTAD/Ag	Spiro-OMeTAD+CuSCN	$CH_3NH_3PbI_{3-x}Cl_x$	7.25 mm^2	12
2016	10.59	FTO/c-TiO_2/ perovskite/spiro-MeOTAD/Ag	Spiro-OMeTAD+F4TCNQ	$CH_3NH_3PbI_{3-x}Cl_x$	0.07 cm^2	13
2015	10.6	FTO/c-TiO_2/Perovskite/Spiro-MeOTAD/Au	Spiro-OMeTAD+Reduced Graphene Oxide (RGO)	$CH_3NH_3PbI_3$	0.06 cm^2	14
2015	9	FTO/c-TiO_2/Perovskite/Spiro-MeOTAD/Au	Spiro-OMeTAD+Decamethylcobaltocene (DMC)/Spiro-OMeTAD/Spiro-OMeTAD+F4TCNQ	$CH3NH_3PbI_{3-x}Cl_x$		15
2015	18.7	FTO/bl-TiO_2/mp-TiO_2/perovskite/ spiro-MeOTAD /Au	po-Spiro-OMeTAD (po-Spiro)+CuPC	$(FAPbI_3)_{0.85}(MAPbBr_3)_{0.15}$	0.0955 cm^2	16

Reference

1. N. Jiang, Z. C. Zheng, C. Qin, R. Liang, Z. Y. Li, Z. Z. Ye and L. P. Zhu, *Ceram Int*, 2023, **49**, 9502.
2. G. Z. Du, L. Yang, C. P. Zhang, X. L. Zhang, N. Rolston, Z. D. Luo and J. B. Zhang, *Adv Energy Mater*, 2022, **12**, 2103966.
3. M. N. Liu, S. Dahlstrom, C. Ahlang, S. Wilken, A. Degterev, A. Matuhina, M. Hadadian, M. Markkanen, K. Aitola, A. Kamppinen, J. Deska, O. Mangs, M. Nyman, P. D. Lund, J. H. Smatt, R. Osterbacka and P. Vivo, *J Mater Chem A*, 2022, **10**, 11721.
4. L. Zhao, J. P. Mou, L. Zhu and J. Song, *J Electron Mater*, 2021, **50**, 6512.
5. J. Liu, W. Z. Liu, E. Aydin, G. T. Harrison, F. H. Isikgor, X. B. Yang, A. S. Subbiah and S. De Wolf, *Acs Appl Mater Inter*, 2020, **12**, 23874.
6. V. Trifiletti, T. Degousee, N. Manfredi, O. Fenwick, S. Colella and A. Rizzo, *Metals-Basel*, 2020, **10**, 14.
7. K. Jiang, F. Wu, G. Y. Zhang, L. N. Zhu and H. Yan, *Sol Rrl*, 2019, **3**, 1900061.
8. W. Luo, C. C. Wu, D. Wang, Z. H. Zhang, X. Qi, X. Guo, B. Qu, L. X. Xiao and Z. J. Chen, *Org Electron*, 2019, **74**, 7.
9. L. Kegelmann, P. Tockhorn, C. M. Wolff, J. A. Marquez, S. Caicedo-Davila, L. Korte, T. Unold, W. Lovenich, D. Neher, B. Rech and S. Albrecht, *Acs Appl Mater Inter*, 2019, **11**, 9172.
10. W. Yu, S. W. Yu, J. Zhang, W. S. Liang, X. L. Wang, X. Guo and C. Li, *Nano Energy*, 2018, **45**, 229
11. J. S. Luo, C. Y. Jia, Z. Q. Wan, F. Han, B. W. Zhao and R. L. Wang, *Journal of Power Sources*, 2017, **342**, 886.
12. M. Li, Z. K. Wang, Y. G. Yang, Y. Hu, S. L. Feng, J. M. Wang, X. Y. Gao and L. S. Liao, *Adv Energy Mater*, 2016, **6**, 1601156.
13. L. K. Huang, Z. Y. Hu, J. Xu, K. Zhang, J. J. Zhang, J. Zhang and Y. J. Zhu, *Electrochim Acta*, 2016, **196**, 328.
14. Q. Luo, Y. Zhang, C. Y. Liu, J. B. Li, N. Wang and H. Lin, *J Mater Chem A*, 2015, **3**, 15996
15. M. C. Jung, S. R. Raga, L. K. Ono and Y. B. Qi, *Sci Rep-Uk*, 2015, **5**, 9863.
16. J. Seo, N. J. Jeon, W. S. Yang, H. W. Shin, T. K. Ahn, J. Lee, J. H. Noh and S. I. Seok, *Adv Energy Mater*, 2015, **5**, 1501320.
